# An NF-kappaB- and IKK-Independent Function of NEMO Prevents Hepatocarcinogenesis by Suppressing Compensatory Liver Regeneration

**DOI:** 10.3390/cancers11070999

**Published:** 2019-07-17

**Authors:** Christiane Koppe, Florian Reisinger, Karina Wehr, Mihael Vucur, Christian Trautwein, Frank Tacke, Mathias Heikenwalder, Tom Luedde

**Affiliations:** 1 Department of Medicine III, University Hospital RWTH Aachen, D-52074 Aachen, Germany; 2 Institute of Virology, Technische Universität München and Helmholtz Zentrum München, 81675 Munich, Germany; 3 Department of Hepatology and Gastroenterology, Charité University Medicine Berlin, D-13353 Berlin, Germany; 4 Division of Chronic Inflammation and Cancer, German Cancer Research Center (DKFZ), D-69120 Heidelberg, Germany

**Keywords:** hepatocarcinogenesis, cholestasis, IKK complex, NF-κB, NEMO, IKKα, IKKβ

## Abstract

The I-κB-Kinase (IKK) complex represents a central signaling nexus in the TNF-dependent activation of the pro-inflammatory NF-κB pathway. However, recent studies suggested that the distinct IKK subunits (IKKα, IKKβ, and NEMO) might withhold additional NF-κB-independent functions in inflammation and cancer. Here, we generated mice lacking all three IKK subunits in liver parenchymal cells (LPC) (IKKα/β/NEMO^LPC-KO^) and compared their phenotype with mice lacking both catalytic subunits (IKKα/β^LPC-KO^), allowing to functionally dissect putative I-κB-Kinase-independent functions of the regulatory subunit NEMO. We show that the additional deletion of NEMO rescues IKKα/β^LPC-KO^ mice from lethal cholestasis and biliary ductopenia by triggering LPC apoptosis and inducing a strong compensatory proliferation of LPC including cholangiocytes. Beyond this beneficial effect, we show that increased hepatocyte cell-death and compensatory proliferation inhibit the activation of LPC-necroptosis but trigger spontaneous hepatocarcinogenesis in IKKα/β/NEMO^LPC-KO^ mice. Collectively, our data show that free NEMO molecules unbound to the catalytic IKK subunits control LPC programmed cell death pathways and proliferation, cholestasis and hepatocarcinogenesis independently of an IKK-related function. These findings support the idea of different functional levels at which NEMO controls inflammation and cancer in the liver.

## 1. Introduction

Hepatocellular carcinoma (HCC) develops in most cases on the basis of chronic inflammation and subsequent liver fibrosis and cirrhosis [1,2]. In advanced HCC, the current treatment strategies can only extend median survival by a few months [2,3], underlining the need for a better functional understanding of key signaling pathways involved in inflammatory liver disease and hepatocarcinogenesis. The nuclear factor (NF)-κB pathway plays a prominent role in inflammatory signaling and is activated in response to cytokines like tumor necrosis factor (TNF). One prominent mediator of NF-κB signaling is the so-called I-κB-Kinase (IKK) complex, consisting of the two catalytic subunits IKKα (also called IKK1) and IKKβ (IKK2), as well as a regulatory subunit called NEMO (IKKγ) [4]. The deregulation of NF-κB signaling is a major contributory factor to the pathophysiology of inflammation [5]. 

Recent experimental studies suggested that the distinct IKK-subunits might have additional, NF-κB-independent functions in hepatic inflammation and cancer [6,7,8,9,10]. As such, the deletion of NEMO in liver parenchymal cells (NEMO^LPC-KO^) caused the spontaneous development of hepatocellular carcinoma driven by apoptotic cell death and compensatory proliferation [7,11]. In contrast, the combined deletion of the catalytic subunits IKKα and IKKβ (IKKα/β^LPC-KO^) also induces apoptotic cell death but impaired the compensatory proliferation of hepatocytes and particularly of intrahepatic biliary cells, thus promoting biliary cell paucity and lethal cholestasis [6,9]. Neither phenotypes occurred in mice lacking NF-κB by deleting all three subunits (RelA, RelB, and c-Rel) in parenchymal liver cells, arguing for NF-κB independent functions of the three IKK subunits [11]. However, up to now, it has remained unclear if the putative function of NEMO in hepatocarcinogenesis is independent of its role in the activation of I-κB-Kinase activity in liver cells. To test this, we generated mice lacking all three IKK complex subunits (IKKα/IKKβ/NEMO^LPC-KO^) and compared their phenotype with IKKα/β^LPC-KO^ mice, which harbor free NEMO molecules unbound to the catalytic IKK subunits. 

## 2. Results

### 2.1. Additional Deletion of Nemo Rescues IKKα/β^LPC-KO^ Mice from Growth Retardation and Lethal Cholestasis

**Figure 1:**
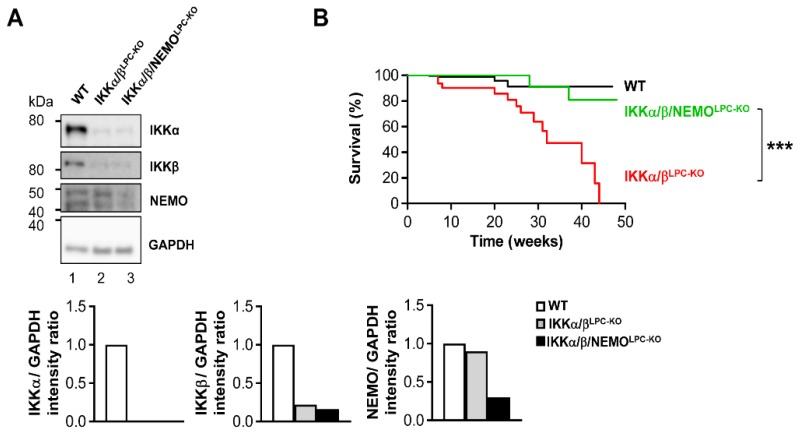

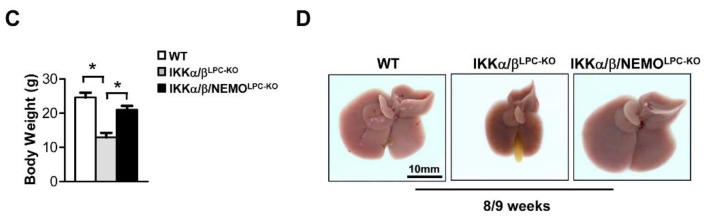
Additional deletion of NEMO in parenchymal liver cells rescues early lethality and growth retardation of IKKα/β^LPC-KO^ mice. (**A**) Immunoblot analysis of IKKα, IKKβ, NEMO and GAPDH (loading control) in whole liver-protein extracts from male wild type (WT), IKKα/β^LPC-KO^ and IKKα/β/NEMO^LPC-KO^ mice with relative intensity ratios. (**B**) Kaplan-Meier curve showing spontaneous death of IKKα/β^LPC-KO^ mice between 23 and 38 weeks of age. *** *p* < 0.001 (WT n = 116, IKKα/β^LPC-KO^ n = 36, and IKKα/β/NEMO^LPC-KO^ n = 41). (**C**) Body weight analysis of male WT, IKKα/β^LPC-KO^ and IKKα/β/NEMO^LPC-KO^ mice. * *p* < 0.05; (n = 4). Results are shown as mean, error bars indicate SEM. (**D**) Representative macroscopic pictures of male WT, IKKα/β^LPC-KO^, and IKKα/β/NEMO^LPC-KO^ livers.

**Figure 2:**
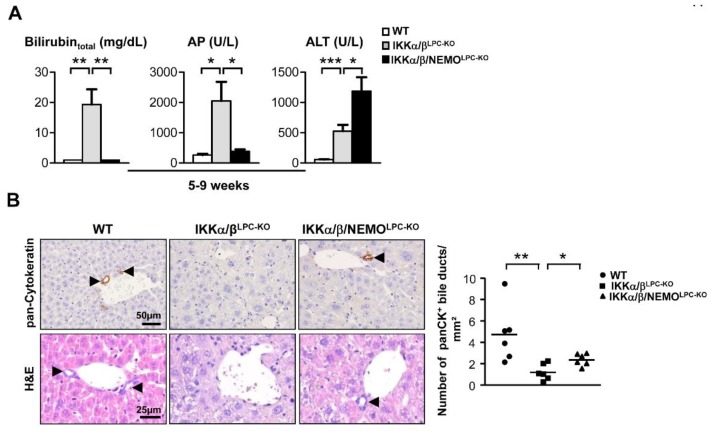
Loss of Nemo expression rescues the cholestasis phenotype of IKKα/β^LPC-KO^ (**A**) Serum level analysis of total bilirubin, alkaline phosphatase (AP) and serum alanine aminotransferase (ALT) in male mice. * *p* < 0.05; ** *p* < 0.01; *** *p* < 0.001 (n = 5). Results are shown as mean, error bars indicate SEM. (**B**) Immunohistochemistry and quantification for pan-Cytokeratin and Haematoxylin and eosin staining (H&E) of liver paraffin sections. * *p* < 0.05; ** *p* < 0.01 (n = 6).

Previous studies demonstrated that the combined deletion of the catalytic subunits IKKα and IKKβ causes severe cholestasis due to the loss of the small intrahepatic bile ducts, thus resulting in an early lethality [6,9]. To unravel the functional role of NEMO molecules unbound to the catalytic IKK subunits in this lethal phenotype, we generated mice with LPC-specific deletion of all three IKK subunits in LPC (IKKα/β/NEMO^LPC-KO^) (Figure 1A). Strikingly, a triple knockout of IKKα, IKKβ and NEMO rescued not only the early lethality seen in IKKα/β^LPC-KO^ animals (Figure 1B), but also the severe growth retardation and very small liver size of these mice (Figure 1C,D). The dramatically reduced body weight and lethality of the IKKα/β^LPC-KO^ mutants correlated with severe cholestasis, reflected by high bilirubin and alkaline phosphatase (AP) levels (Figure 2A). Of note, the additional deletion of NEMO suppressed not only the development of cholestasis in IKKα/β/NEMO^LPC-KO^ animals (Figure 2A) but went along with elevated numbers of small periportal bile duct cells in the triple mutant mice (Figure 2B). Interestingly, alanine transaminase (ALT) levels were significantly increased in IKKα/β/NEMO^LPC-KO^ mice compared to IKKα/β^LPC-KO^ double mutants (Figure 2A). In summary, the additional deletion of NEMO in IKKα/β^LPC-KO^ mice rescued these animals from lethal cholestasis and growth retardation, but increased ALT values as a surrogate for hepatitis and liver cell death. 

### 2.2. Free NEMO Molecules Correlate with Increased Necroptosis of Parenchymal Liver Cell, but Low Levels of Apoptosis

**Figure 3:**
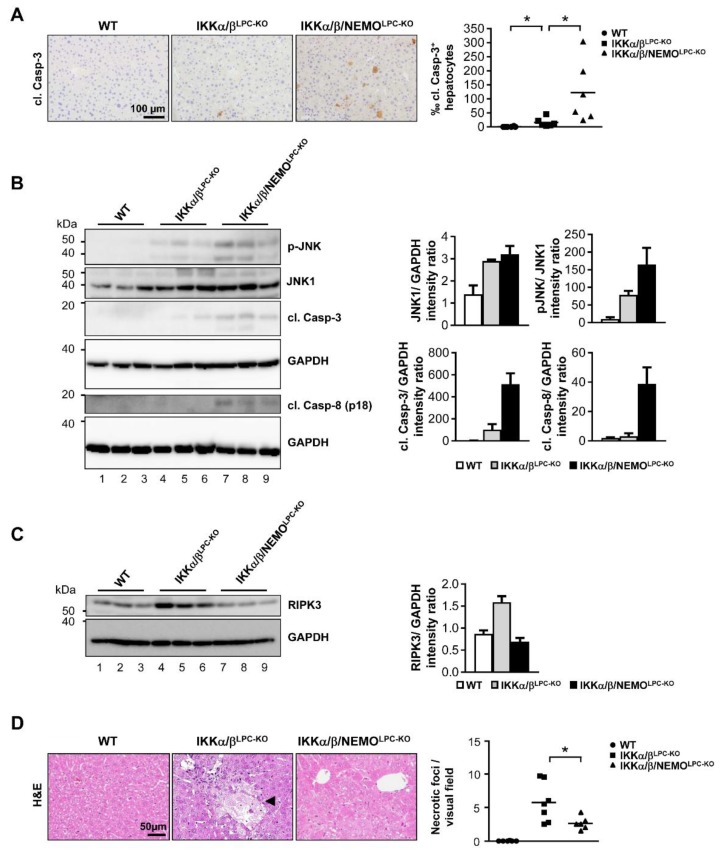
Nemo deletion increases apoptotic death and inhibits necroptosis in livers of IKKα/β deleted mice. (**A**) Immunohistochemistry and quantification of liver paraffin sections for cl. Casp-3. * *p* < 0.05 (n = 6). (**B**) Immunoblot analysis of cleaved Caspase-8 (cl. Casp-8), cleaved Caspase-3 (cl. Casp-3), p-JNK, JNK1 and GAPDH (loading control) in whole liver-protein extracts from male WT, IKKα/β^LPC-KO^ and IKKα/β/NEMO^LPC-KO^ mice with relative intensity ratios added. (**C**) Immunoblot analysis of RIKP3 and GAPDH (loading control) in whole liver-protein extracts from male WT, IKKα/β^LPC-KO^ and IKKα/β/NEMO^LPC-KO^ mice with relative intensity ratios. (**D**) H and E staining of liver paraffin sections of WT, IKKα/β^LPC-KO^ and IKKα/β/NEMO^LPC-KO^ mice and quantification of necrotic foci. * *p* < 0.05 (n = 6–7).

Based on the strong effects of free NEMO (unbound to catalytic IKK subunits) on ALT levels, we further examined potential NEMO-specific effects on LPC cell death as a fundamental driver of liver diseases, and particularly HCC [12]. First, we performed electrophoretic mobility shift assay (EMSA) analysis to confirm that a triple knockout of all IKK subunits did not trigger any paradox activation of NF-κB (Supplementary Figure S1). Next, we examined the effects of additional NEMO deletion on the spontaneous activation of cell death in IKKα/β^LPC-KO^ mice. In line with previous results [6,9], we detected a significant upregulation of apoptotic cell death in the liver tissue of IKKα/β^LPC-KO^ mice compared to wild type (WT) mice by immunohistochemical staining of cleaved Caspase-3 (Figure 3A). Interestingly, cleavage of Caspase-3 was notably increased by the additional deletion of NEMO (Figure 3A). In line, Western blot analysis revealed also more spontaneous cleavage of Caspase-8 and Caspase-3 as well as an increased phosphorylation of JNK in the livers of triple mutant IKKα/β/NEMO^LPC-KO^ mice (Figure 3B), correlating with the previously shown rise in serum ALT levels in IKKα/β/NEMO^LPC-KO^ animals (see Figure 2A). 

We further investigated whether free NEMO molecules unbound to the catalytic IKK subunits triggered necroptotic cell death in IKKα/β^LPC-KO^ mice. By Western blot analysis, we found that elevated expression levels of RIPK3 (as surrogates for necroptotic cells [13]) in liver tissue of IKKα/β^LPC-KO^ animals were strikingly reduced in livers of IKKα/β/NEMO^LPC-KO^ mice (Figure 3C). This finding correlated with a significant reduction of intrahepatic necrotic foci in IKKα/β/NEMO^LPC-KO^ animals (Figure 3D). Taken together, these findings illustrate that free NEMO molecules unbound to the catalytic IKK subunits prevent apoptosis but promote necroptotic cell death of liver parenchymal cells. 

### 2.3. Additional Deletion of Nemo Rescues IKKα/β^LPC-KO^ Mice from Cholestasis, but Triggers Hepatocarcinogenesis

**Figure 4:**
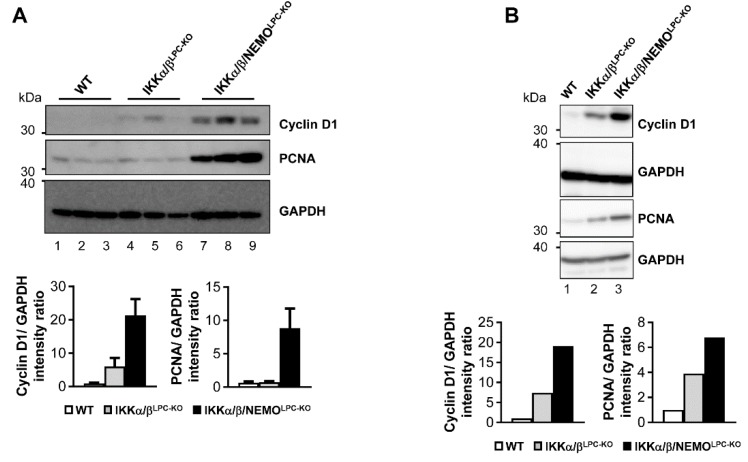
Enhancement of compensatory proliferation in IKKα/β/NEMO^LPC-KO^ mice. (**A** and **B**) Immunoblot analysis of Cyclin D1, PCNA and GAPDH (loading control) in three whole liver- (**A**) and primary hepatocyte- (**B**) protein extracts from male WT, IKKα/β^LPC-KO^ and IKKα/β/NEMO^LPC-KO^ mice and relative intensity ratios.

**Figure 5:**
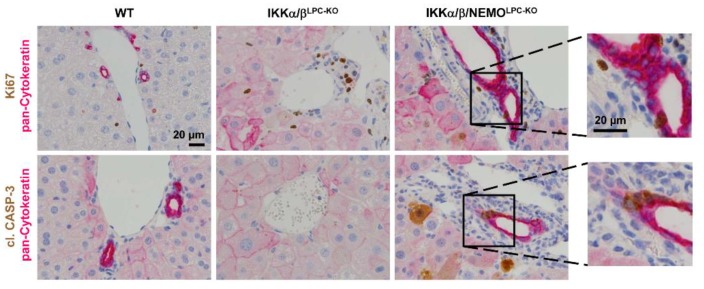
Increased regeneration of the biliary compartment in IKKα/β^LPC-KO^ mice by additional deletion of *NEMO.* Representative immunohistochemical staining of liver sections from WT, IKKα/β^LPC-KO^ and IKKα/β/NEMO^LPC-KO^ mice (n = 3). (Upper panel: pan-Cytokeratin is stained pink/red and Ki67 is stained brown; lower panel: pan-Cytokeratin is stained pink/red and cl. Caspase-3 is stained brown).

We finally tested if free NEMO molecules influenced compensatory LPC proliferation as a potential mechanism underlying the rescue of triple mutant animals from cholestasis. Strikingly, hepatocyte proliferation was significantly increased in IKKα/β/NEMO^LPC-KO^ mice compared to double mutant mice, which was shown by Western blot for Cyclin D1 and PCNA on whole liver extracts as well as freshly isolated primary hepatocytes (Figure 4A,B). Interestingly, we could not only detect increased hepatocyte proliferation in IKKα/β/NEMO^LPC-KO^ animals but also proliferating cholangiocytes demonstrated by the double stainings for Ki-67 and pan-Cytokeratin (Figure 5). These data confirm the hypothesis that ductopenia in IKKα/β^LPC-KO^ animals is functionally linked with a defective regenerative response of biliary cells in a setting of chronic hepatic inflammation (Supplementary Figure S2) and that this defective regenerative response can be overcome by the additional deletion of NEMO.

**Figure 6:**
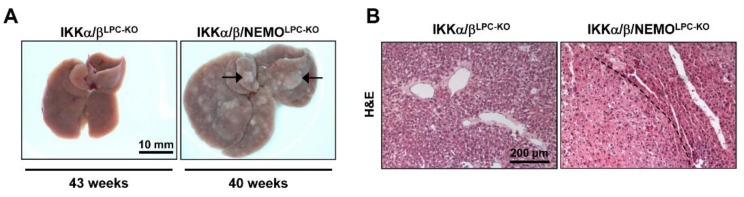
NEMO deletion induces hepatocarcinogenesis in mice lacking liver parenchymal IKKα/β expression. (**A**) Representative macroscopic pictures of 37–43 weeks-old IKKα/β^LPC-KO^, and IKKα/β/NEMO^LPC-KO^ livers (n = 5). (**B**) H and E staining of liver paraffin sections of old IKKα/β^LPC-KO^ and IKKα/β/NEMO^LPC-KO^ mice.

Finally, IKKα/β/NEMO^LPC-KO^ mice from 37 weeks of age showed multiple hepatic tumors on the macroscopic and histological level, whereas age-matched IKKα/β^LPC-KO^ mice did not show any signs of tumor development (Figure 6A,B). In line, the expression of Gp73, which is highly expressed in hepatocellular carcinoma cells [14], was massively upregulated in liver tissue of old IKKα/β/NEMO^LPC-KO^ mice (Supplementary Figure S1). Together, these data provide evidence for distinct functions of NEMO molecules unbound to the catalytic IKK subunits in cell death and regeneration of liver parenchymal cell.

## 3. Discussion

In the last decade, the perception of the IKK complex and its subunits in liver signaling and pathology has profoundly changed. Originally considered as part of a linear signaling cascade mediating the activation of NF-κB, recent studies, including our own, provided evidence that IKK subunits exhibit important cellular functions beyond their contribution to NF-κB activation [6,7,9,10,11,15]. Specifically, the molecule NEMO appears to control additional pathways that play a fundamental role in basic biological processes, such as cell death and proliferation. Moreover, NEMO mutations are critically involved in human diseases like incontinentia pigmenti and anhidrotic ectodermal dysplasia with immunodeficiency [16,17]. In addition, it was demonstrated that NEMO expression is lost in up to 40% of HCC and that low NEMO expression correlated with a poor 5-year overall survival [18]. This together highlights the pathophysiological relevance of NEMO in human disease. 

In this study, we focused on the biological and pathophysiological functions of free NEMO molecules unbound to the catalytic IKK subunits in parenchymal liver cells. This I-κB-Kinase-independent setting allowed us not only to examine systematically the autonomous functions of NEMO, but also to explore the impact of a deletion of the whole IKK complex on liver homeostasis and pathogenesis, which to the best of our knowledge has not been examined previously in any other organ in vivo. By this approach, we could show that IKKα/β and NEMO regulate LPC cell death and compensatory liver regeneration in functionally distinct ways. As such, the additional deletion of NEMO in the IKKα/β^LPC-KO^ mice model boosted the apoptotic death but prevented necroptosis of liver parenchymal cells. This switch in cell death modes resulted in a strong net increase in the compensatory proliferation of hepatocytes and cholangiocytes. While this increased LPC proliferation could trigger a sufficient regeneration and architectural restoration of the biliary system to rescue mice from the lethal cholestasis seen in IKKα/β^LPC-KO^ mice, it was associated with cancer development in IKKα/β/NEMO^LPC-KO^ animals. The fact that apoptosis but not necroptosis was associated with a strong compensatory proliferation appears counter-intuitive, since necrosis is generally considered to the more “reactive” cell death form compared to apoptosis [12]. While this paradox is not fully understood yet, we could previously demonstrate by genetic sub-crossing approaches in mice lacking TAK1 in LPC that in these animals, apoptosis but not necroptosis represented a major trigger of compensatory LPC proliferation, specific chromosomal aberration patterns and malignant transformation of hepatocytes [13]. 

The crucial biological effects of distinct cell death modes in the phenotype of IKKα/β/NEMO^LPC-KO^ mice underline that free NEMO molecules unbound to the catalytic IKK subunits can directly control the activation of RIP Kinase dependent signaling pathways. In particular, RIPK1 represents a central signal node in cell death, inflammation, and tumorigenesis [19,20,21]. As such, it was suggested that NEMO can inhibit apoptosis by preventing the formation of a RIPK1/FADD/Caspase-8 complex [11]. Another study suggested that TBK1 and IKKε prevent cell death by RIPK1 phosphorylation and that the recruitment of these two non-canonical IKKs is mediated by NEMO [22]. In our previous study, we showed that additional deletion of RIPK1 in IKKα/β^LPC-KO^ mice does not significantly influence spontaneous apoptotic liver cell death [9]. This argues for the presence of RIPK1-independent apoptosis in the liver tissue of IKKα/β^LPC-KO^ mice. Interestingly, in the present study, the deletion of NEMO in IKKα/β^LPC-KO^ mice causes a significant boost of apoptotic cell death. A possible explanation could be an activation of RIPK1-dependent apoptosis in IKKα/β^LPC-KO^ livers by ablation of NEMO, which would support the concept of NEMO as an inhibitor of RIPK1-dependent apoptosis.

How exactly free NEMO molecules in IKKα/β^LPC-KO^ mice promoted LPC necroptosis is less clear, but it was suggested in previous in vitro studies that NEMO promoted necroptosis by exerting a scaffolding function in the formation of the RIPK1/RIPK3 necrosome and contributing to TNFα-induced mitochondrial dysfunction [23,24]. 

Collectively, our approach of a triple knockout of all distinct IKK subunits in LPC allowed us to genetically and functionally dissect a specific role of free NEMO molecules in controlling programmed cell death, compensatory proliferation of LPC, which showed to be fundamental for the control of cholestasis and hepatocarcinogenesis. In the light of previous genetic studies, our present data provide further evidence against the previous notion of the NF-κB axis as a one-dimensional pathway towards a highly complex functional network with multiple alternative functions of key regulatory mediators like NEMO or RIP Kinases. The fact that these alternative functions are specifically regulated by posttranscriptional mechanisms like ubiquitination and phosphorylation opens the possibility for novel pharmacological inhibitors allowing specific targeting of these functions in inflammation and cancer. On the other hand, our findings illustrate that a specific targeting of the pro-inflammatory function of NF-κB — a pathway that is activated in most HCC [25] — could be revitalized as a chemo-preventive strategy in chronic human liver disease, if these strategies are specific enough not to attenuate the anti-carcinogenic effects of NEMO or RIPK1. 

## 4. Methods

### 4.1. Generation and In Vivo Treatments of Genetically Modified Mouse Models

Mice carrying loxP-site-flanked (floxed) alleles of IKKα/IKBKA (IKKα^FL^) [26] and IKKβ/IKBKB (IKKβ^FL^) [27] were crossed to Alfp-Cre transgenic mice [28] to generate a liver parenchymal cell-specific knockout (LPC) of both genes. Mice with double-knockout of *Ikkα/Ikkβ* (IKKα/β^LPC-KO^) and NEMO (NEMO^LPC-KO^) [10] were crossed to generate a triple-knockout of IKKα/IKKΒ/NEMO (IKKα/β/NEMO^LPC-KO^) in LPC. In all experiments, littermates carrying the respective loxP-flanked alleles but lacking expression of Cre recombinase were used as wild type (WT) controls. Mice were bred on a mixed C57/BL6xSV129Ola genetic background. Only sex-matched animals were compared. Unless otherwise indicated, mice were analyzed at 7 to 9 weeks of age. All animals in this study were treated in full compliance with the guidelines for animal care approved by the institutional German Animal Care Committee (LANUV, Recklinghausen, Germany; Az.: 84-02.04.2016.A267; permission date: 6 December 2016). 

Liver injury experiments were performed on 6 weeks old mice. LPS (Sigma) was administered i.p. at a concentration of 2.5 μg/g body weight. 

### 4.2. Isolation and Stimulation of Primary Hepatocytes

Primary hepatocytes were isolated from mouse livers and cultured as previously described [6].

### 4.3. Western Blot and Immunoblot Analysis

Protein lysates were isolated from liver samples as described previously [9]. Proteins were separated by SDS-polyacrylamide gel electrophoresis (SDS-PAGE), transferred to PVDF membrane and analyzed by immunoblot with the following antibodies: α-NEMO, α-β-actin (Sigma Aldrich, St. Louis, MO, USA), α-cleaved Caspase-3, α-cleaved Caspase-8, α-IKKβ, p-JNK, JNK1 (Cell Signaling Technology, Danvers, MA, USA), α-IKKα, α-RIPK3 (Novus Biologicals, Centennials, CO, USA), α-PCNA (Thermo Fisher Scientific, Waltham, MA, USA), and α-GAPDH (Bio-Rad Laborories, Hercules, CA, USA). As secondary antibodies, α-rabbit-HRP, and α-mouse-HRP (GE Healthcare, Little Chalfont, UK) were used. Densitometry was performed by using ImageJ software (ImageJ, NIH, Bethesda, MD, USA).

### 4.4. Electrophoretic Mobility Shift Assay (EMSA)

Nuclear extracts were prepared from whole livers and assayed as described [6] with a double-stranded, 32P-labeled oligonucleotide containing an NF-κB consensus site (5′-CGG GCT GGG GAT TCC CCA TCT CGG TAC-3′). 

### 4.5. Histological and Immunohistochemical Stainings

Liver tissue was fixed in 4% paraformaldehyde and paraffin embedded. Paraffin sections (2 μm) were stained with hematoxylin and eosin (H&E) or various primary and secondary antibodies. Automated immunohistochemical stainings, image acquisition and quantification were performed as previously described [9]. The following antibodies were used: α-pan-Cytokeratin (Dako A/S; 1:300), α-Ki67 (NeoMarkers, Fremont, CA, USA; 1:200), α-cleaved Caspase-3 (Cell Signaling Technology, Danvers, MA, USA; 1:300), and F4/80 (BMA Biomedicals AG, 1:120, Augst, Switzerland). 

### 4.6. Serum Analysis

Serum ALT, AST, AP activities and total serum bilirubin levels were determined by standard procedures in the Institute of Clinical Chemistry of the RWTH University Hospital Aachen.

### 4.7. Statistics

Results are expressed as the mean + standard error of the mean (SEM). The significance of differences between groups was assessed using unpaired two-sample t-Test. Survival curves were generated using the Kaplan-Meier method and the significance of the difference in survival rate was determined by the log-rank test (GraphPad Prism; GraphPad SoftwareInc., La Jolla, CA, USA).

## 5. Conclusions

We generated mice lacking all IKK subunits (IKKα, IKKβ, and NEMO) in parenchymal liver cells and compared the phenotype to mice lacking only the catalytic subunits IKKα and IKKβ to functionally assess the potential role of free NEMO molecules unbound to the catalytic subunits in the mediation of cholestasis and liver cancer. With this approach, we could demonstrate that NEMO has NF-κB- and IKK-independent function in promoting necroptosis and preventing apoptosis in parenchymal liver cells. 

This molecular function is crucial for the regulation of biliary architecture and liver cancer, underlining that NEMO can control liver inflammation and hepatocarcinogenesis on different functional levels.

## Supplementary Materials

The following are available online at https://www.mdpi.com/2072-6694/11/7/999/s1, Figure S1, Analysis of NF-κB DNA-binding activity, Figure S2, Analysis of F4/80 positive cell infiltrates, Figure S3, Increased GP73 expression in mice lacking the IKK complex expression.
