# Supplementary material for: An NF-kappaB- and IKK-Independent Function of NEMO Prevents Hepatocarcinogenesis by Suppressing Compensatory Liver Regeneration
Source: Cancers (Basel). 2019 Jul 17;11(7):999. doi: 10.3390/cancers11070999 (PMC6678501; doi:10.3390/cancers11070999)
Supplement: Supplementary file 1 [file cancers-11-00999-s001.pdf]

cancers MDPI

Supplementary Material

An NF-kappaB- and IKK-Independent Function of NEMO Prevents Hepatocarcinogenesis by Suppressing Compensatory Liver Regeneration

Christiane Koppe, Florian Reisinger, Karina Wehr, Mihael Vucur, Christian Trautwein, Frank Tacke, Mathias Heikenwalder and Tom Luedde

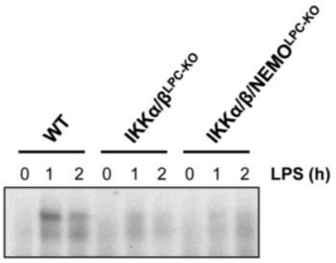

Figure S1: Analysis of NF-κB DNA-binding activity
Electrophoretic mobility shift assay (EMSA) on whole liver extracts from WT, IKKα/β^{LPC-KO} and IKKα/β/NEMO^{LPC-KO} mice treated with LPS for different time points.

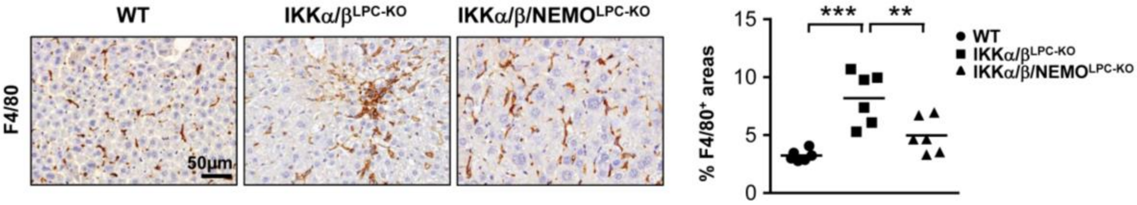

Figure S2: Analysis of F4/80 positive cell infiltrates
Immunohistochemistry and quantification of liver paraffin sections stained for F4/80. ***P*<0.01; ****P*<0.001 (n=6).

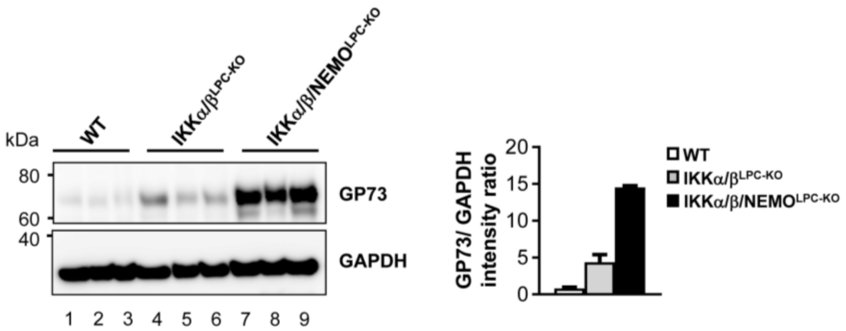

Figure S3: Increased GP73 expression in mice lacking the IKK complex expression
Western blot analysis of whole protein extracts from male WT, IKKα/β^{LPC-KO} and IKKα/β/NEMO^{LPC-KO} mice against GP73 and GAPDH (loading control) with relative intensity ratio.

Figure 1A

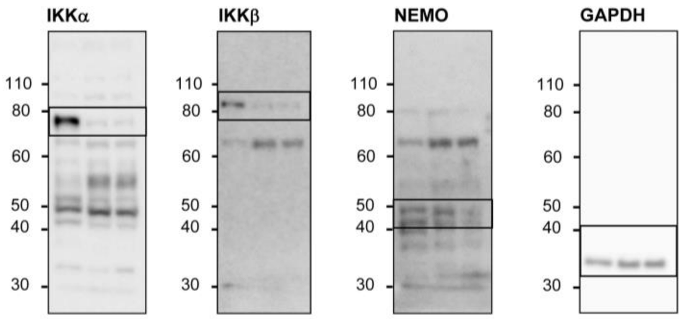

Figure 3B

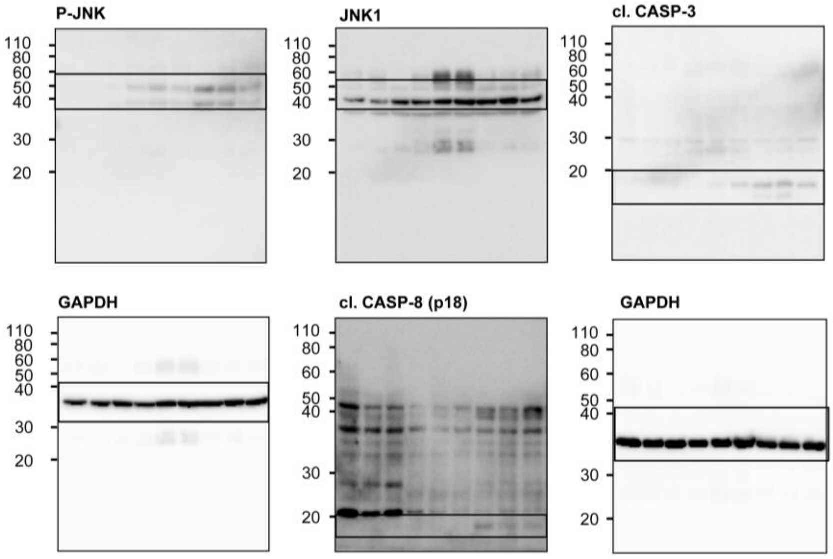

Figure 3C

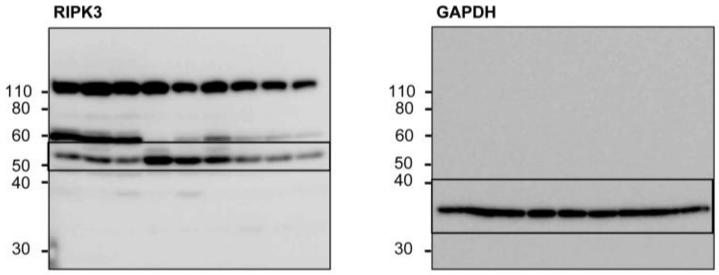

Western Blot for Figures 1 and 3.

Figure 4A

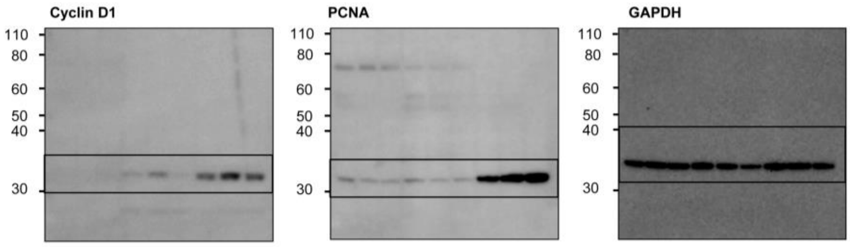

Figure 4B

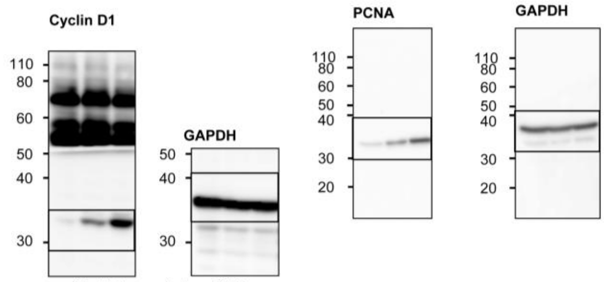

The blot was cut above 50kDa.

Figure S3

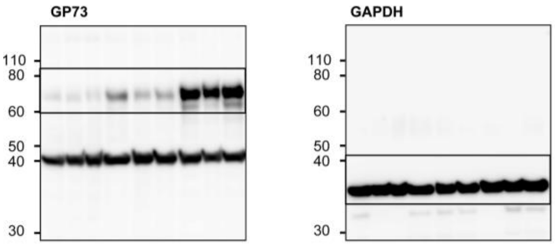

Western Blot for Figures 4 and S3.
